# Development and validation of a robust immune-related prognostic signature in early-stage lung adenocarcinoma

**DOI:** 10.1186/s12967-020-02545-z

**Published:** 2020-10-07

**Authors:** Pancheng Wu, Yi Zheng, Yanyu Wang, Yadong Wang, Naixin Liang

**Affiliations:** 1 Department of Thoracic Surgery, Peking Union Medical College Hospital, Peking Union Medical College, Chinese Academy of Medical Sciences, Beijing, 100730 China; 2 Department of Oncology, The Second Affiliated Hospital of Xi’an Jiaotong University, Xi’an, 710004 China; 3 Department of Thoracic Surgery, Peking Union Medical College Hospital, Chinese Academy of Medical Sciences, Beijing, 100730 China

**Keywords:** Lung adenocarcinoma, Overall survival, Immune-related genes, Prognosis, Risk score

## Abstract

**Background:**

The incidence of stage I and stage II lung adenocarcinoma (LUAD) is likely to increase with the introduction of annual screening programs for high-risk individuals. We aimed to identify a reliable prognostic signature with immune-related genes that can predict prognosis and help making individualized management for patients with early-stage LUAD.

**Methods:**

The public LUAD cohorts were obtained from the large-scale databases including 4 microarray data sets from the Gene Expression Omnibus (GEO) and 1 RNA-seq data set from The Cancer Genome Atlas (TCGA) LUAD cohort. Only early-stage patients with clinical information were included. Cox proportional hazards regression model was performed to identify the candidate prognostic genes in GSE30219, GSE31210 and GSE50081 (training set). The prognostic signature was developed using the overlapped prognostic genes based on a risk score method. Kaplan–Meier curve with log-rank test and time-dependent receiver operating characteristic (ROC) curve were used to evaluate the prognostic value and performance of this signature, respectively. Furthermore, the robustness of this prognostic signature was further validated in TCGA-LUAD and GSE72094 cohorts.

**Results:**

A prognostic immune signature consisting of 21 immune-related genes was constructed using the training set. The prognostic signature significantly stratified patients into high- and low-risk groups in terms of overall survival (OS) in training data set, including GSE30219 (HR = 4.31, 95% CI 2.29–8.11; *P* = 6.16E−06), GSE31210 (HR = 11.91, 95% CI 4.15–34.19; *P* = 4.10E−06), GSE50081 (HR = 3.63, 95% CI 1.90–6.95; *P* = 9.95E−05), the combined data set (HR = 3.15, 95% CI 1.98–5.02; *P* = 1.26E−06) and the validation data set, including TCGA-LUAD (HR = 2.16, 95% CI 1.49–3.13; *P* = 4.54E−05) and GSE72094 (HR = 2.95, 95% CI 1.86–4.70; *P* = 4.79E−06). Multivariate cox regression analysis demonstrated that the 21-gene signature could serve as an independent prognostic factor for OS after adjusting for other clinical factors. ROC curves revealed that the immune signature achieved good performance in predicting OS for early-stage LUAD. Several biological processes, including regulation of immune effector process, were enriched in the immune signature. Moreover, the combination of the signature with tumor stage showed more precise classification for prognosis prediction and treatment design.

**Conclusions:**

Our study proposed a robust immune-related prognostic signature for estimating overall survival in early-stage LUAD, which may be contributed to make more accurate survival risk stratification and individualized clinical management for patients with early-stage LUAD.

## Background

Lung cancer is the leading cause of death from cancer. In the United States, there will be approximately 228,820 newly diagnosed cases and 135,720 deaths in 2020 [1]. Lung adenocarcinoma (LUAD) is the most common histological type and accounts for nearly 60% of non-small cell lung cancer (NSCLC), which comprises approximately 85% of lung cancer [2–4]. Surgical lobectomy remains the preferred treatment strategy for patients with operable early-stage LUAD [5]. Although patients with early-stage LUAD have a relatively superior prognosis, nearly 10–44% of these patients still die within 5 years after surgical intervention [6, 7]. Recently, several studies revealed that adjuvant chemotherapy brought a clear 5-year survival benefit ranged from 4 to 10% for patients with resected stage II LUAD and can be considered for stage IB LUAD with primary tumor more than 4 cm [8–11], but not for patients with stage IA because of the potential detrimental effect [12]. Thus, besides the traditional clinical factors, it is imperative to develop a novel prognostic signature to perform personalized survival risk stratification and identify the high-risk early-stage patients who might benefit from additional systemic therapy.

In recent years, numerous studies have reported prognostic signatures to make survival stratification and predict prognosis for patients with LUAD using genomics and transcriptomics data [13–15]. Unfortunately, the signatures proposed by these studies have not been incorporated into clinical practice owing to the problems such as small sample size and insufficient independent validation [16, 17]. Nowadays, the available public, large-scale databases containing enough gene expression data, such as TCGA (The Cancer Genome Atlas) and GEO (Gene Expression Omnibus) database, bring the opportunity to make more reliable prognostic signatures for lung cancer [18]. Immune system has been shown to play a crucial role in cancer initiation and progression [19, 20]. In addition, avoiding immune destruction has been accepted as a novel hallmark of cancer [21]. Recently, immunotherapies have achieved a notably and durable response in LUAD by targeting specific immune checkpoints like PD-1 or PD-L1 [22, 23]. Several studies have reported immune-related gene signatures which could predict prognosis and provide potential targets for immunotherapy in patients with LUAD [24–26]. However, few prognostic models have focused on immune-related genes in early-stage LUAD.

In this study, we used the gene expression data sets from GEO and TCGA to develop and validate a prognostic prediction model for early-stage LUAD based on immune-related genes. A novel 21-gene based prognostic immune signature with robust prediction power for early-stage LUAD was developed, which allows clinicians to evaluate the prognosis of patients with early-stage LUAD and might provide promise for individualized therapeutic interventions.

## Methods

### Data preprocessing

We downloaded four independent NSCLC microarray data sets from GEO database (https://www.ncbi.nlm.nih.gov/geo/) using the GEOquery package [27]. Only early-stage LUAD patients were included. Patients without survival status or whose overall survival time shorter than 30 days were removed from the study. Among these data sets, the gene expression data of GSE30219 [28], GSE31210 [29, 30] and GES50081 [31] were generated by the same platform GPL570 (Affymetrix Human Genome U133 Plus 2.0 Array). These data sets were defined as training set and selected to screen for the candidate prognostic genes, while GSE72094 [32], another microarray data set, was chose for independent validation. Besides, The gene expression data and corresponding clinical information of TCGA-LUAD cohort, a RNA-seq data set, were downloaded by the UCSC Xena platform [33, 34], which was used for another independent validation. The general information of these datasets was summarized in Additional file 1: Table S1. The gene expression data of GEO and TCGA-LUAD data sets were normalized by the limma and DESeq2 package, respectively [35, 36]. Overall, a total of 1091 patients were enrolled in our study, including 82 patients from GSE30219, 204 patients from GSE31210, 127 patients from GSE50081, 311 patients from GSE72094 and 367 patients from TCGA-LUAD. The baseline characteristics of the patients enrolled in the study were described in Additional file 2: Table S2.

### Development of the prognostic gene signature

**Fig. 1:**
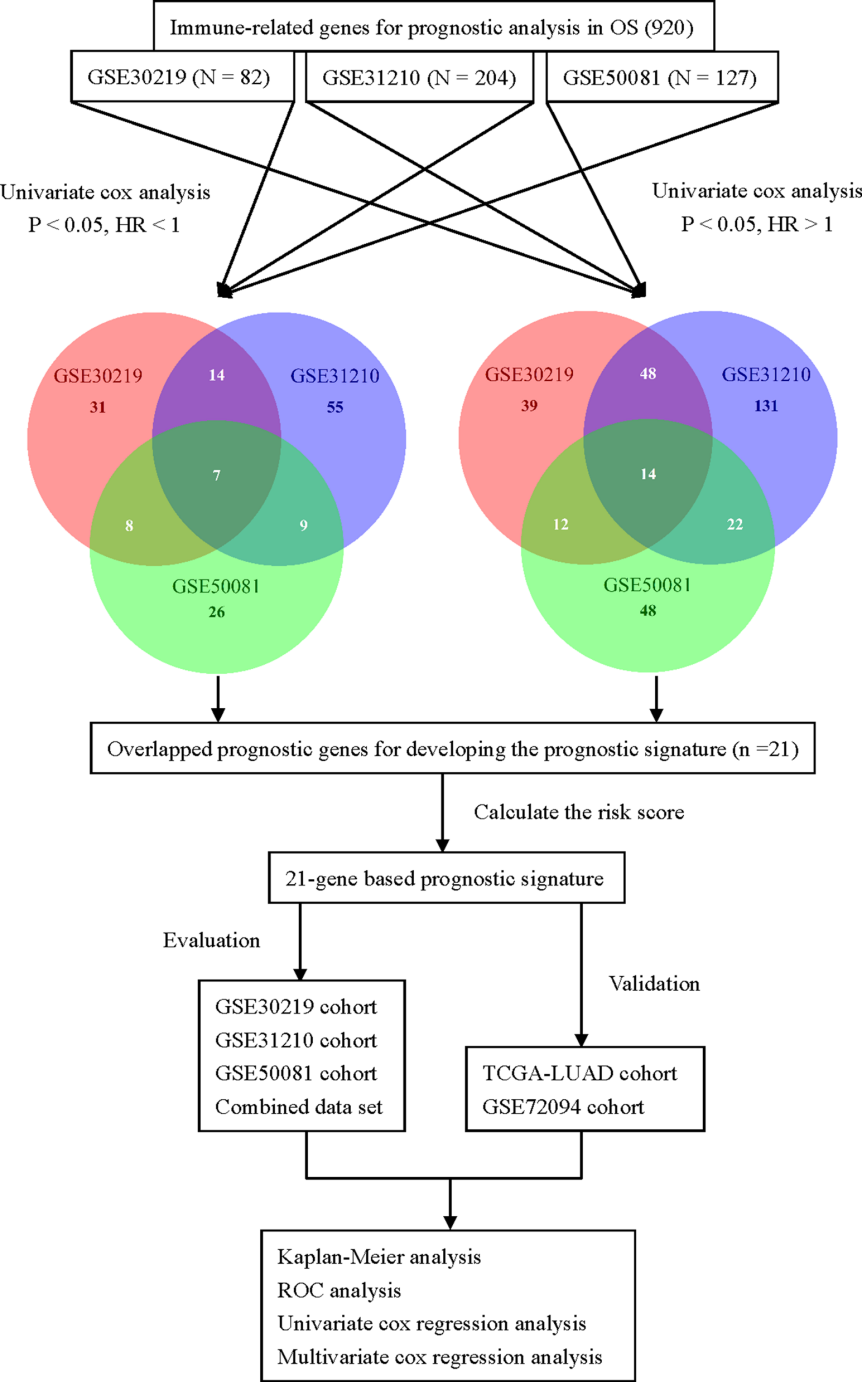
Flow chart of this study

We constructed the prognostic gene signature by focusing on the immune-related genes, which were downloaded from the InnateDB database (https://innatedb.com/) [37]. The list of the immune-related genes was summarized in Additional file 3: Table S3. The flow chart of this study was presented in Fig. 1. Firstly, univariate cox proportional hazards regression model was performed to screen for the candidate prognostic genes (*p* < 0.05) associated with OS in GSE30219, GSE31210 and GSE50081 cohort, respectively. Candidate genes with Hazard ratio (HR) > 1 were considered as risky prognostic genes, while HR < 1 as protective prognostic genes. The overlapped candidate prognostic genes were selected to develop the prognostic signature based on risk score model. In addition, the three microarray data sets were merged into 1 combined data set for further analysis.

Then a risk score for each patient was established based on a linear combination of the overlapped candidate prognostic genes expression levels weighted by the regression coefficient (*β*) derived from the univariate cox regression analysis [38, 39]. The risk score formula was defined as the following:

$${\text{Risk score }} = \mathop \sum \limits_{i = 1}^{n} exp_{i} * \beta_{i}$$

The n, *exp*_*i*_ and *β*_*i*_ in the above formula represent the number of prognostic genes, the expression value and the coefficient of gene *i,* respectively [40]. Optimal cutoff value of the risk score in each data set was determined by the survminer package in R [41]. According to the cutoff value, patients were classified into high- and low-risk groups.

### Evaluation of the immune-related prognostic signature

To assess the prognostic value of this prognostic signature, we firstly estimated the survival curves between the high- and low-risk groups by the Kaplan–Meier method using the survival and survminer package in GSE30219, GSE31210, GSE50081 and the combined data set, respectively [41, 42], with log-rank test to determine the statistical significances in OS between two groups. Meanwhile, time-dependent receiver operating characteristic (ROC) curve was conducted to evaluate the performance of this signature by calculating the area under the ROC curves (AUC) using timeROC package [43]. Furthermore, the same risk score formula was employed on GSE72094 and TCGA-LUAD cohort, which were served as independent validation data sets, to further evaluate and validate the efficiency of this signature.

### Functional annotation and enrichment analysis

To acquire the potential biological processes of the overlapped prognostic genes, Gene Ontology (GO) enrichment and Kyoto Encyclopedia of Genes and Genomes (KEGG) pathway analysis were performed using clusterProfiler package [44].

### Statistical analysis

All statistical analyses were performed using R (version 3.6.2; R Foundation for Statistical Computing) [45] and RStudio (version 1.2.1335) (https://rstudio.com/). To investigate whether the gene signature was an independent prognostic factor for early-stage LUAD, univariate analysis was performed to evaluate the association of the gene signature and other clinical parameters with overall survival. Risk factors (*p* < 0.2) derived from univariate analysis were selected for further analysis in multivariate cox regression model [46, 47]. Heatmap was generated using the pheatmap package [48]. The detailed information of the system, software and packages using in the study were summarized in Additional file 4: Table S4. *p* < 0.05 deemed statistically significant.

## Results

### Identification of 21 immune-related prognostic genes in the training set

A total of 1091 patients with early-stage LUAD (533 men [49%], 558 women [51%]; median age [range], 66 [30–89] years), including 413 patients in the training set and 678 patients in the validation set, were enrolled in the analysis. Among 1051 immune-related genes from the innateDB database, 920 genes were measured in the training set. Under the cutoff value of *p* < 0.05, 173 genes in GSE30219, 300 genes in GSE31210 and 146 genes in GSE50081 were identified as candidate prognostic genes which were significantly associated with OS. After overlapping these prognostic genes among these data sets, 21 overlapped genes were finally screened, including 14 risky genes and 7 protective genes. The general information of the overlapped genes and corresponding coefficients were summarized in Additional file 5: Table S5.

### Development of the 21-gene based immune-related prognostic signature

We calculated the 21-gene based risk score for each patient in the training set using the risk score formula (Additional file 6: Table S6). Patients were classified into high- and low-risk groups using the optimal cutoff analyzed by the survminer package. The cutoff value in each cohort was summarized in Additional file 7: Table S7. The distribution of the risk scores, survival status and the expression levels of the 21 genes in the training set were shown in Additional file 8: Figure. S1.

**Fig. 2:**
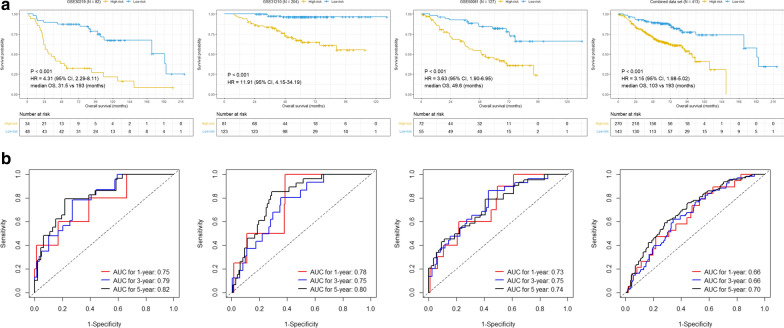
Correlation between the 21-gene signature and Overall survival in the training set (early-stage LUAD). **a** Kaplan–Meier survival curves between high- and low-risk groups, **b** ROC curves for 1-year, 3-year and 5-year survival prediction by the 21-gene signature

Kaplan–Meier survival curves revealed that patients in the high-risk group shown significantly poorer OS than patients in the low-risk group (Fig. 2a). Moreover, the AUCs for 1-year, 3-year and 5-year were 0.75, 0.80 and 0.82 in GSE30219, 0.78, 0.75 and 0.81 in GSE31210 and 0.73, 0.75 and 0.74 in GSE50081, respectively (Fig. 2b), suggesting that this 21-gene signature achieved a relatively high performance for early-stage LUAD survival prediction. Furthermore, we conducted survival analysis in the combined data set to assess the reliability of this signature. Consistent with the results of single data set in the training set, Kaplan–Meier curve showed that patients in the high-risk group exhibited shorter OS than those in the low-risk group (*p* < 0.0001) (Fig. 2a). The AUCs for 1-year, 3-year and 5-year were 0.66, 0.66 and 0.70, respectively (Fig. 2b), implying that this gene signature also had a good performance for prognosis prediction in the combined data set.

### External validation of the 21-gene prognostic signature

**Fig. 3:**
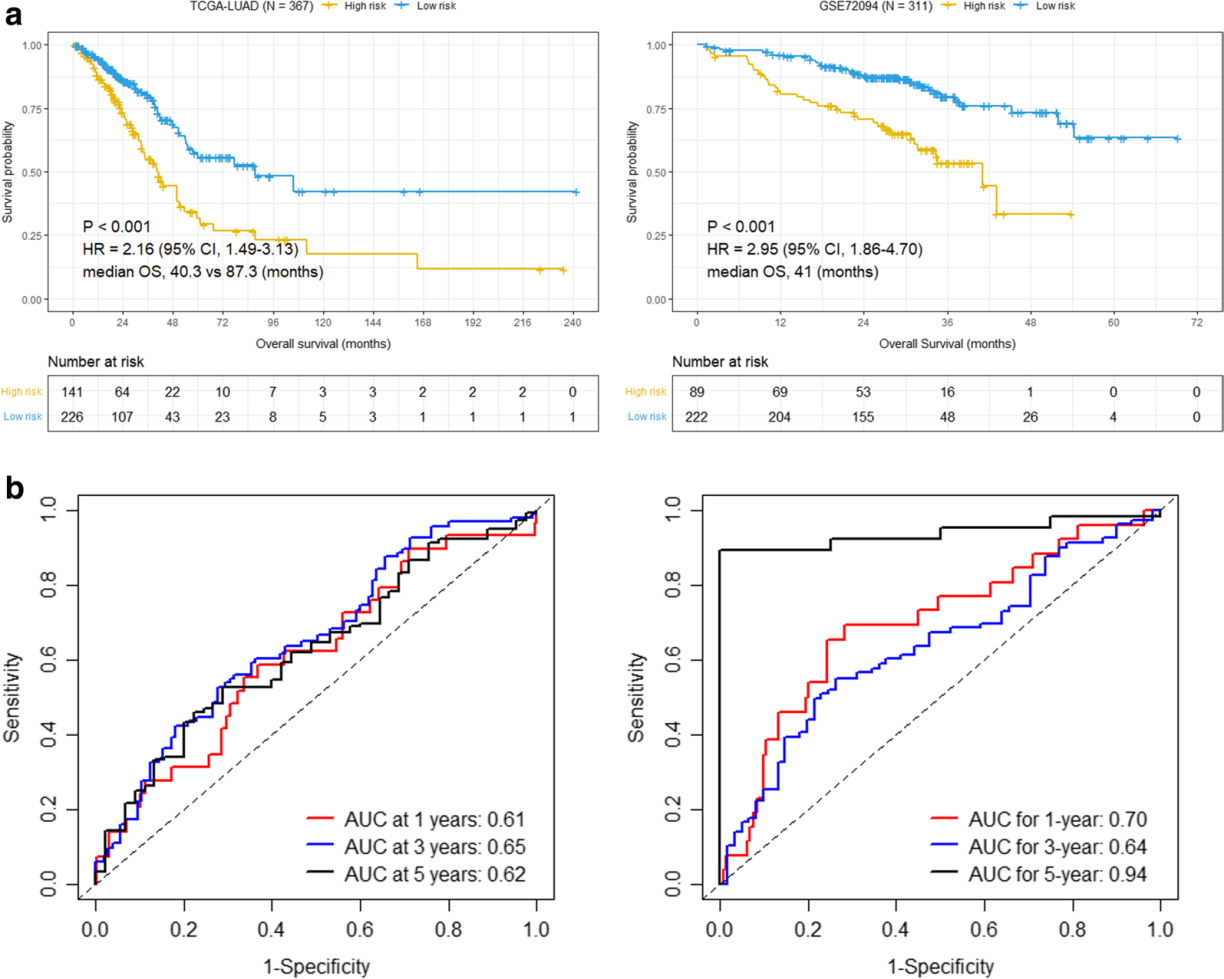
Correlation between the 21-gene signature and Overall survival in the validation set (early-stage LUAD). **a** Kaplan–Meier survival curves between high- and low-risk groups in TCGA-LUAD and GSE72094 cohort, respectively. **b** ROC curves for 1-year, 3-year and 5-year survival prediction by the 21-gene signature in TCGA-LUAD and GSE72094 cohort, respectively

To further validate the robustness of the 21-gene signature, the risk score for each patient was calculated using the same risk score formula in two independent data sets, including TCGA-LUAD and GSE72094 cohort. We divided the patients into high- and low-risk group according to the optimal cutoff. Consistent with the results in the training set, patients of high-risk group shown conspicuously poorer OS than those of low-risk group in both TCGA-LUAD and GSE72094 cohort (*p* < 0.0001, Fig. 3a). The AUCs for 1-year, 3-year and 5-year were 0.61, 0.66 and 0.62 in TCGA-LUAD, and 0.70, 0.64 and 0.94 in GSE72094 (Fig. 3b), which implies that the prognostic signature has a valid performance for OS prediction in validation data sets. The distribution of the risk scores, survival status and the expression levels of the 21 genes in the validation set were shown in Additional file 8: Figure. S1. The data for Kaplan–Meier survival analysis and ROC analysis were summarized in Additional file 9: Table S8. Taken together, these results suggest that this 21-gene based prognostic signature is robust in prognosis prediction for early-stage LUAD and can be used in both microarray and RNA-sequencing data sets.

### The 21-gene prognostic signature is an independent prognostic factor

Univariate and multivariate cox analysis were performed in both training and validation sets to investigate whether this 21-gene prognostic signature could be served as an independent prognostic factor for patients with early-stage LUAD. The prognostic signature and other available clinicopathological factors were included for analysis. Univariate regression analysis indicated that the prognostic signature was significantly associated with OS for early-stage LUAD in GSE30219 (HR = 4.31, 95% CI 2.29–8.11, *P* = 6.16E−06), GSE31210 (HR = 11.91, 95% CI 4.15–34.19; *P* = 4.10E−06), GSE50081 (HR = 3.63, 95% CI 1.90–6.95; *P* = 9.95E−05), combined data set (HR = 3.15, 95% CI 1.98–5.02; *P* = 1.26E−06) (Table 1), TCGA-LUAD (HR = 2.16, 95% CI 1.49–3.13; *P* = 4.54E−05) and GSE72094 (HR = 2.95, 95% CI 1.86–4.70; *P* = 4.79E−06) (Table 2). Then, risk factors (*P* < 0.2) derived from the univariate analysis were selected for further multivariate analysis. The results shown that there was a significantly association between the prognostic signature and OS in GSE30219 (HR = 5.01, 95% CI 2.50–10.06; *P* = 5.75E−06), GSE31210 (HR = 8.82, 95% CI 2.86–27.14; *P* = 1.48E−04), GSE50081 (HR = 2.74, 95% CI 1.37–5.46; *P* = 4.24E−03), combined data set (HR = 3.01, 95% CI 1.86–4.85; *P* = 6.44E−06) (Table 1), TCGA-LUAD (HR = 1.91, 95% CI 1.28–2.85; *P* = 1.55E−03) and GSE72094 (HR = 2.94, 95% CI 1.81–4.79; 1.47E−05) (Table 2). These results demonstrated that the 21-gene based prognostic signature was an independent prognostic factor for patients with early-stage LUAD in both training set and validation set after adjusting for other clinical and pathologic factors.

**Table 1:** Univariate and multivariate Cox regression analyses of the 21-gene signature and OS in the training set (early-stage LUAD)

Variables		Patients (N)	Univariate analysis			Multivariate analysis		
			HR	95% CI	*p* value	HR	95% CI	*p* value
GSE30219								
Age	> 65/ ≤ 65	23/59	1.765	0.946–3.295	7.42E−02	2.36	1.179–4.724	1.53E−02
Gender	Male/female	64/18	1.148	0.532–2.478	7.24E−01			
T stage	T2/T1	12/69	2.046	1.019–4.107	4.40E−02	0.982	0.352–1.94	9.63E−01
Risk score	High/low	34/48	4.308	2.287–8.114	6.16E−06	5.012	2.498–10.058	5.75E−06
GSE31210								
Age	> 65/ ≤ 65	47/157	2.779	1.349–5.724	5.59E−03	3.172	1.445–6.964	4.01E−03
Gender	Male/female	95/109	1.686	0.818–3.476	1.57E−01	1.033	0.347–3.078	9.54E−01
Stage	II/I	42/462	4.297	2.092–8.828	7.21E−05	2.444	1.152–5.186	1.99E−02
Smoking	Yes/no	99/105	1.908	0.918–3.966	8.35E−02	1.726	0.565–5.271	3.38E−01
ALK fusion	±	7/197	1.057	0.144–7.777	9.56E−01			
EGFR mutation	±	116/88	0.46	0.222–0.956	3.76E−02	1.658	0.512–5.375	3.99E−01
KRAS mutation	±	19/185	0.992	0.3–3.282	9.89E−01			
Triple negative	Yes/no	62/142	0.436	0.212–0.894	2.34E−02	0.543	0.174–1.698	2.94E−01
MYC copy	High/low	16/187	0.767	0.183–3.22	0.716942752			
Risk score	High/low	81/123	11.914	4.151–34.193	4.10E−06	8.817	2.864–27.14	1.48E−04
GSE50081								
Age	> 65/ ≤ 65	87/40	1.455	0.774–2.735	2.44E−01			
Gender	Male/female	65/62	1.41	0.807–2.463	2.28E−01			
Stage	II/I	35/92	2.443	1.383–4.316	2.10E−03	1.905	1.068–3.399	2.90E−02
T stage	T2/T1	82/30	2.435	1.214–4.883	1.22E−02	1.625	0.785–3.365	1.91E−01
Smoking	Yes/no	92/23	1.682	0.75–3.776	2.07E−01			
Risk score	High/low	72/55	3.632	1.897–6.953	9.95E−05	2.736	1.373–5.455	4.24E−03
Combined data set								
Age	> 65/ ≤ 65	157/256	2.314	1.621–3.304	3.82E−06	2.414	1.686–3.456	1.49E−06
Gender	Male/female	224/189	1.574	1.081–2.291	1.80E−02	1.582	1.084–2.307	1.73E−02
Stage	II/I	90/323	2.79	1.934–4.026	4.08E−08	2.065	1.42–3.004	1.49E−04
Risk score	High/low	270/143	3.153	1.981–5.016	1.26E−06	3.005	1.863–4.846	6.44E−06

**Table 2:** Univariate and multivariate Cox regression analyses of the 21-gene signature and OS in the validation set (early-stage LUAD)

Variables		Patients (N)	Univariate analysis			Multivariate analysis		
			HR	95% CI	*p* value	HR	95% CI	*p* value
GSE72094								
Age	> 65/ ≤ 65	219/92	1.638	0.952–2.817	7.47E−02	1.807	1.042–3.135	3.52E−02
Gender	Male/female	141/170	1.449	0.917–2.289	1.12E−01	1.386	0.871–2.207	1.69E−01
Stage	II/I	65/246	2.137	1.313–3.479	2.24E−03	2.258	1.38–3.695	1.20E−03
Smoking	Yes/no	237/26	1.334	0.482–3.696	5.79E−01			
EGFR mutation	Yes/no	35/276	0.092	0.013–0.659	1.76E−02	0.137	0.019–1.004	5.05E−02
KRAS mutation	Yes/no	105/206	1.701	1.075–2.691	2.33E−02	1.554	0.976–2.475	6.33E−02
TP53 mutation	Yes/no	77/234	1.461	0.893–2.392	1.31E−01	1.154	0.69–1.932	5.86E−01
STK11 mutation	Yes/no	48/263	0.799	0.41–1.559	5.11E−01			
Risk score	High/low	89/222	2.952	1.857–4.695	4.79E−06	2.942	1.806–4.793	1.47E−05
TCGA-LUAD								
Age	> 65/ ≤ 65	190/168	1.308	0.897–1.907	1.62E−01	1.513	1.018–2.249	4.05E−02
Gender	Male/female	168/199	1.069	0.739–1.546	7.25E−01			
Stage	II/I	113/254	2.607	1.795–3.785	4.84E−07	2.186	1.444–3.309	2.19E−04
T stage	T2/T1	201/141	1.423	0.942–2.147	9.35E−02	1.131	0.775–1.813	4.34E−01
Smoking	Yes/no	273/49	0.923	0.54–1.579	7.70E−01			
Risk score	High/low	141/226	2.16	1.492–3.128	4.54E−05	1.909	1.279–2.849	1.55E−03

### Prognosis prediction by combining the 21-gene prognostic signature with stage

**Fig. 4:**
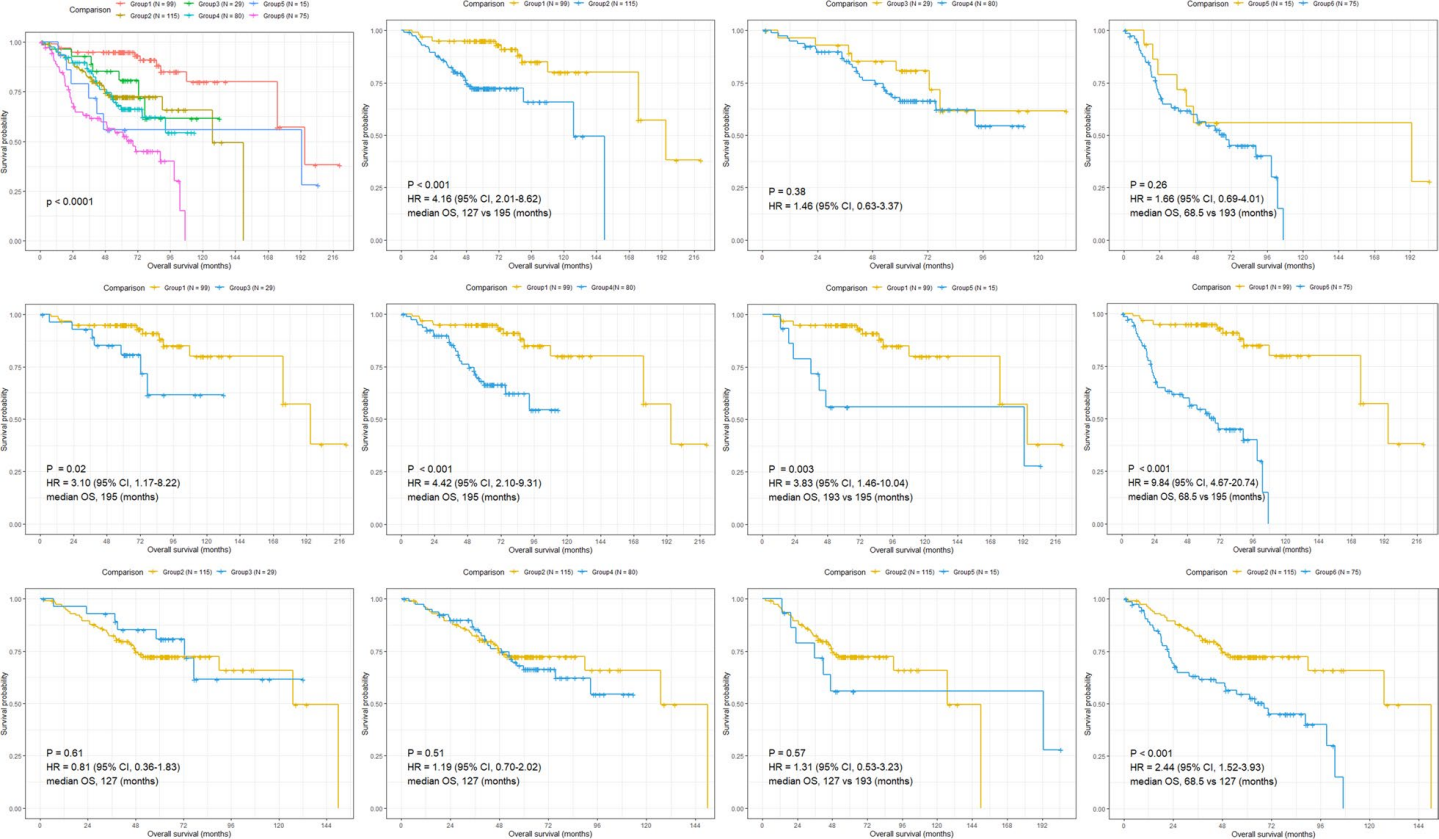
Kaplan–Meier curves of overall survival for patients grouped by stage and 21-gene signature combination (early-stage LUAD)

Multivariate analysis revealed that the prognostic signature and stage were both independent prognostic factors in the combined data set, suggesting a complementary value. Therefore, we attempted to develop an integrated prognostic model for survival prediction by combining the prognostic signature with tumor stage in the combined data set. Based on the risk and stage, patients were classified into six groups: group 1 (stage IA with low-risk), group 2 (stage IA with high-risk), group 3 (stage IB with low-risk), group 4 (stage IB with high-risk), group 5 (stage II with low-risk) and group 6 (stage II with high-risk) (Fig. 4). Kaplan–Meier survival analysis were performed between different groups. The results revealed that patients in group 2, group3, group 4, group5 and group 6 had worse prognosis compared with patients in group 1, with group 1 exhibited the best prognosis and group 6 showed the worst (Fig. 4). Nevertheless, there was no significant difference between patients in group 2 and group 3/4/5 (Fig. 4). These results display that patients of stage IA with high-risk have similar prognosis to those of stage IB and stage II with low-risk, suggesting adjuvant chemotherapy might be beneficial for stage IA LUAD with high-risk. Additionally, patients of early-stage LUAD could be divided into six different groups based on the stage and prognostic signature, which might be a more precise scheme to predict prognosis for patients with early-stage LUAD in the future practice.

### Functional annotation and enrichment analysis of the 21-gene prognostic signature

**Fig. 5:**
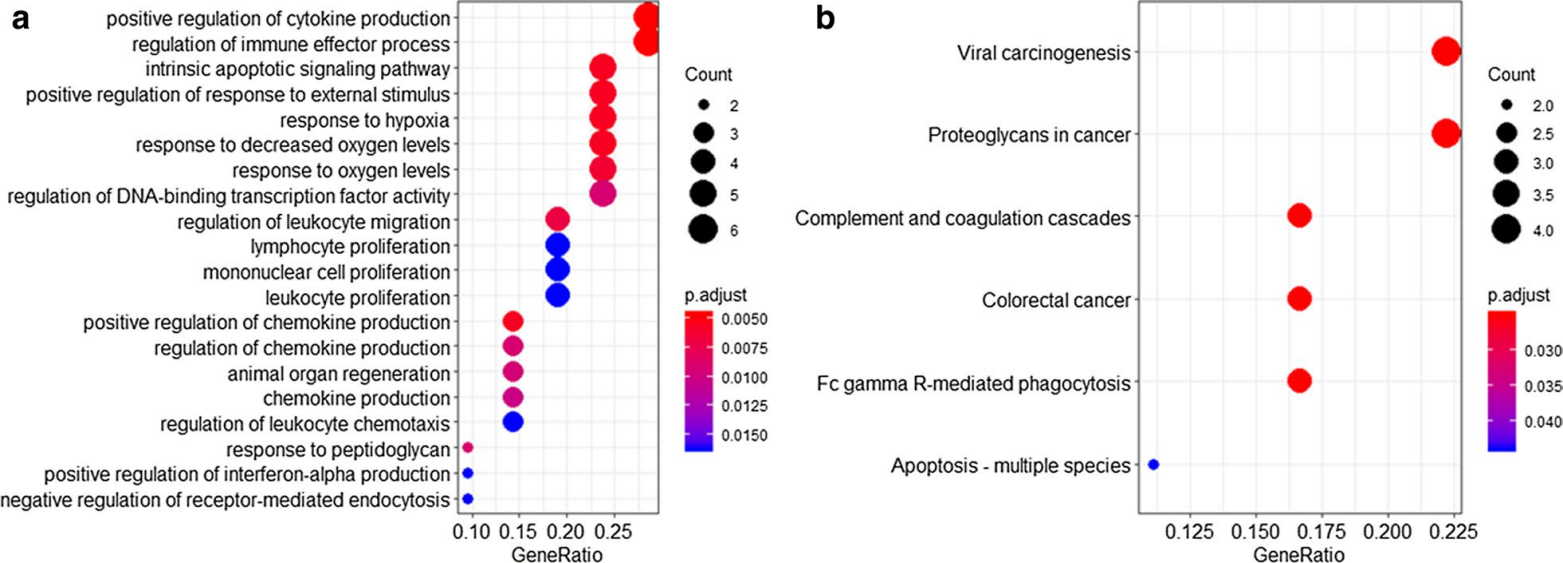
Functional enrichment analysis of the 21 prognostic genes. **a** Gene Ontology analysis, **b** Kyoto Encyclopedia of Genes and Genomes pathway analysis

To identify the underlying biological processes and pathways within this 21-gene signature, we performed GO enrichment and KEGG pathway analysis. The results indicated these genes were mainly enriched in biological processes such as positive regulation of cytokine production (GO:0001819), regulation of immune effector process (GO:0002697) and intrinsic apoptotic signaling pathway (GO:0097193) (Fig. 5). In addition, KEGG analysis revealed that several pathways like viral carcinogenesis, proteoglycans in cancer and Fc-gamma R-mediated phagocytosis (Fig. 5) were enriched among these genes.

## Discussion

Previous studies have reported different prognostic biomarkers for patients with early-stage LUAD [28, 49–51]. However, none of these studies focused on the immune-related genes in prognosis prediction. Recently, several studies have proposed prognostic signatures using immune-related genes for LUAD [24–26]. Nevertheless, some concerns hamper the prediction power of these prognostic signatures, such as insufficient sample size, lack of external independent validation or effective validation. In the present study, we developed a novel prognostic signature based on 21 immune-related genes for early-stage LUAD and validated it in two independent cohorts. Our prognostic signature was significantly associated with OS for early-stage LUAD and could further identify the high- and low-risk early-stage LUAD patients with significant differences in OS. Besides, the 21-gene prognostic signature showed a good prediction performance in all enrolled studies including GEO and TCGA-LUAD data sets, suggesting that our signature had a cross-platform compatibility. Multivariate regression analysis revealed that the 21-gene prognostic signature was an independent prognostic factor for all enrolled studies. These results suggest that the 21-gene prognostic signature could effectively predict the overall survival for early-stage LUAD.

In the aera of immunotherapy, it may hold great promise to discover prognostic and predictive biomarkers that are related to tumor immune microenvironment, which can be used for identifying novel molecular targets for patients [52]. Our functional enrichment analysis suggests that the genes in our prognostic signature are widely involved in the immune process. Among all the 21 prognostic genes, 14 (e.g., *AQP3*, *BIRC5*, *C5AR1*, *HMOX1*, *IL32*, *IL6ST*, *MIF*, *MMP12*, *PLAUR*, *PMAIP1*, *RAC1*, *SMAD6*, *SPHK1*, *USP7*) have been reported to be served as a prognostic biomarker or suggested to be a novel therapeutic targets for lung adenocarcinoma [53–67]. The remaining 7 genes, including *ARF6*, *C7*, *ELF4*, *ITPR1*, *MOV10*, *PTCH1* and *RIPK2*, have not been previously reported to be associated with LUAD prognosis and might act as potential biomarker. We were particularly interested in studying *ARF6*, which was a member of small GTPases ADP-ribosylation factor family, and its downstream effector AMAP1 have been reported overexpressed in several types of cancer and could promote cancer cell proliferation, invasion and migration [68–71]. For example, *KRAS* and *TP53* oncogenes could promote PD-L1 recycling and cell surface expression through *ARF6-AMAP1* pathway, which is significantly involved in the immune evasion of pancreatic ductal adenocarcinoma cells [72].

Currently, tumor staging system has been widely used for prognosis prediction and treatment design for LUAD. However, prognosis might vary in patients with same stage owing to the variabilities in clinical behavior caused by genomic changes [73, 74]. Thus, it is critically needed to develop reliable prognostic biomarkers to predict prognosis and help clinical oncologists optimally select early-stage patients who might obtain survival benefit from additional system therapy. In the integrated prediction model analysis, early-stage LUAD patients could be stratified into six different groups by combining our 21-gene prognostic signature with tumor stage. Besides, no statistical significance exists in prognosis between stage IA patients with high-risk and stage II patients with low-risk. These findings may help clinicians identify high-risk patients and make individualized treatment design for these patients.

The limitations in our study need to be noted. First, although different cohorts from GEO and TCGA databases have been included in our study to develop and validate the immune-related prognostic signature, the study presents a retrospective design. Future large-scale prospective clinical studies needed to confirm our findings. Second, the data of specific mutations such as *EGFR*, *KRAS* and *TP53* were only available in GSE31210 and GSE72094 cohort, thus it might be insufficient to assess the 21-gene prognostic signature with the specific mutations. Finally, the biological mechanisms of these prognostic genes in early-stage LUAD and the association of the prognostic signature with several prognostic biomarker such as PD-L1, IL-7R [75], CD8^+^ [76], are still unknown, Future studies are required to explore and clarify molecular functions of these immune-related prognostic genes during early-stage LUAD progression and the association between these genes with above prognostic biomarkers.

## Conclusions

In summary, we developed and validated a promising immune-related prognostic signature comprising of 21 immune-related genes, which could serve as an independent prognostic biomarker for OS prediction in early-stage LUAD. Furthermore, a prediction model by combining our prognostic signature with tumor stage could more accurately evaluate patient’s prognosis. These findings might provide novel therapeutic targets and be used for making individualized management and hold promise for improving survival for patients with early-stage LUAD.

## Supplementary information

**Additional file 1: Table S1.** The general information of the enrolled datasets.

**Additional file 2: Table S2.** The baseline characteristics of patients included in this study.

**Additional file 3: Table S3.** The list of the immune-related genes downloaded from the InnateDB database.

**Additional file 4: Table S4.** The detailed information of the system, software and packages using in this study.

**Additional file 5: Table S5.** The general information of the overlapped prognostic genes and corresponding coefficients.

**Additional file 6: Table S6.** The specific risk score formula using in this study.

**Additional file 7: Table S7.** The cutoff values for each data set.

**Additional file 8: Figure S1.** The distribution of the risk scores, survival status and gene expression levels in the enrolled data set.

**Additional file 9: Table S8.** The data for Kaplan-Meier survival analysis and ROC analysis.

## Data Availability

The data sets analyzed in this study are available on the public databases.

## Abbreviations

LUAD: Lung adenocarcinoma
GEO: Gene Expression Omnibus
TCGA: The Cancer Genome Atlas
ROC: Receiver operating characteristic
OS: Overall survival
NSCLC: Non-small cell lung cancer
HR: Hazard ratio
AUC: Area under the curve
GO: Gene Ontology
KEGG: Kyoto Encyclopedia of Genes and Genomes
