# Supplementary material for: Development and validation of a robust immune-related prognostic signature in early-stage lung adenocarcinoma
Source: J Transl Med. 2020 Oct 7;18:380. doi: 10.1186/s12967-020-02545-z (PMC7542703; doi:10.1186/s12967-020-02545-z)
Supplement: Supplementary file 4 — Additional file 4: Table S4. The detailed information of the system, software and packages using in this study. [file 12967_2020_2545_MOESM4_ESM.docx]

| Datasets | Downloaded date | Version |
| --- | --- | --- |
| GSE30219 | Jan 02, 2020 | Submission date Jun 26, 2011, Last update date Mar 25, 2019 |
| GSE31210 | Dec 12, 2019 | Submission date Aug 04, 2011, Last update date Mar 25, 2019 |
| GSE50081 | Jan 11, 2020 | Submission date Aug 21, 2013, Last update date Mar 25, 2019 |
| GSE72904 | Jan 28, 2020 | Submission date Aug 14, 2015, Last update date May 07, 2018 |
| TCGA-LUAD | Aug 29, 2019 | Jul 20, 2019 |

**1. The downloaded date of the data.**

**2. The version of the system and software details.**

platform x86_64-w64-mingw32

arch x86_64

os mingw32

system x86_64, mingw32

status

major 3

minor 6.2

year 2019

month 12

day 12

svn rev 77560

language R

version.string R version 3.6.2 (2019-12-12)

nickname Dark and Stormy Night

**3. The version of the packages using in the study.**

| Name | Version |
| --- | --- |
| clusterprofiler | 3.14.3 |
| cowplot | 1.0.0 |
| Data.table | 1.12.8 |
| DESeq | 1.38.0 |
| DESeq2 | 1.26.0 |
| dplyr | 0.8.5 |
| edgeR | 3.28.1 |
| GEOquery | 2.54.1 |
| ggplot2 | 3.3.0 |
| ggpubr | 0.3.0 |
| pheatmap | 1.0.12 |
| stringr | 1.4.0 |
| survival | 3.1-12 |
| tidyr | 1.0.2 |
| timeROC | 0.4 |
| Venndiagram | 1.6.20 |
| surviminer | 0.4.3 |
| readr | 1.3.1 |
| readxl | 1.2.0 |
