# Supplementary material for: Development and validation of a robust immune-related prognostic signature in early-stage lung adenocarcinoma
Source: J Transl Med. 2020 Oct 7;18:380. doi: 10.1186/s12967-020-02545-z (PMC7542703; doi:10.1186/s12967-020-02545-z)
Supplement: Supplementary file 5 — Additional file 5: Table S5. The general information of the overlapped prognostic genes and corresponding coefficients. [file 12967_2020_2545_MOESM5_ESM.docx]

| **The general information of the overlapped 21 prognostic genes** | | | | |
| --- | --- | --- | --- | --- |
| **Ensembl ID** | **Gene symbol** | **Chromosome** | **Gene type** | **Coefficient** |
| ENSG00000187555 | USP7 | 16: 8,892,097-8,975,328 | Protein coding | -1.1249563 |
| ENSG00000176170 | SPHK1 | 17: 76,376,584-76,387,860 | Protein coding | 0.6378106 |
| ENSG00000137834 | SMAD6 | 15: 66,702,236-66,782,849 | Protein coding | -0.5930002 |
| ENSG00000104312 | RIPK2 | 8: 89,757,806-89,791,064 | Protein coding | 0.7625178 |
| ENSG00000136238 | RAC1 | 7: 6,374,527-6,403,967 | Protein coding | 3.1401212 |
| ENSG00000185920 | PTCH1 | 9: 95,442,980-95,517,057 | Protein coding | -0.4780358 |
| ENSG00000141682 | PMAIP1 | 18: 59,899,948-59,904,306 | Protein coding | 0.3924924 |
| ENSG00000011422 | PLAUR | 19: 43,646,095-43,670,547 | Protein coding | 0.5700375 |
| ENSG00000155363 | MOV10 | 1: 112,673,141-112,700,746 | Protein coding | 0.7387577 |
| ENSG00000262406 | MMP12 | 11: 102,862,736-102,874,982 | Protein coding | 0.1862029 |
| ENSG00000240972 | MIF | 22: 23,894,383-23,895,227 | Protein coding | 0.9523012 |
| ENSG00000150995 | ITPR1 | 3: 4,493,345-4,847,840 | Protein coding | -0.604526 |
| ENSG00000134352 | IL6ST | 5: 55,935,095-55,995,022 | Protein coding | -0.7451425 |
| ENSG00000008517 | IL32 | 16: 3,065,297-3,082,192 | Protein coding | 0.455947 |
| ENSG00000100292 | HMOX1 | 22: 35,380,361-35,394,207 | Protein coding | 0.6355181 |
| ENSG00000102034 | ELF4 | X:130,064,874-130,110,716 | Protein coding | 1.0035687 |
| ENSG00000112936 | C7 | 5: 40,909,497-40,984,643 | Protein coding | -0.1911605 |
| ENSG00000197405 | C5AR1 | 19: 47,290,023-47,322,066 | Protein coding | 0.3863955 |
| ENSG00000089685 | BIRC5 | 17: 78,214,186-78,225,636 | Protein coding | 0.367417 |
| ENSG00000165527 | ARF6 | 14: 49,893,082-49,897,054 | Protein coding | 1.1668938 |
| ENSG00000165272 | AQP3 | 9: 33,441,156-33,447,596 | Protein coding | -0.3042632 |
