# Supplementary material for: Development and validation of a robust immune-related prognostic signature in early-stage lung adenocarcinoma
Source: J Transl Med. 2020 Oct 7;18:380. doi: 10.1186/s12967-020-02545-z (PMC7542703; doi:10.1186/s12967-020-02545-z)
Supplement: Supplementary file 6 — Additional file 6: Table S6. The specific risk score formula using in this study. [file 12967_2020_2545_MOESM6_ESM.docx]

**Risk Score Formula**

**Risk score =** exp**_(_**_USP7)_ * (-1.1249563) + exp_(SPHK1)_ * 0.6378106 + exp_(SMAD6)_ * (-0.5930002) + exp_(RIPK2_

_)_ * 0.7625178 + exp_(RAC1)_ * 3.1401212 + exp_(PTCH1)_ * (-0.4780358) + exp_(PMAIP1)_ * 0.3924924 + exp_(PLAUR_

_)_ * 0.5700375 + exp_(MOV10)_ * 0.7387577 + exp_(MMP12)_ * 0.1862029 + exp_(MIF)_ * 0.9523012 + exp_(ITPR1)_ * (-0.604526) + exp_(IL6ST)_ * (-0.7451425) + exp_(IL32)_ * 0.455947 + exp_(HMOX1)_ * 0.6355181 + exp_(ELF4)_ * 1.0035687 + exp_(C7)_ * (-0.1911605) + exp_(C5AR1)_ * 0.3863955 + exp_(BIRC5)_ * 0.367417 + exp_(ARF6)_ * 1.1668938 + exp_(AQP3)_ * (-0.3042632)
