# Supplementary material for: Development and validation of a robust immune-related prognostic signature in early-stage lung adenocarcinoma
Source: J Transl Med. 2020 Oct 7;18:380. doi: 10.1186/s12967-020-02545-z (PMC7542703; doi:10.1186/s12967-020-02545-z)
Supplement: Supplementary file 7 — Additional file 7: Table S7. The cutoff values for each data set. [file 12967_2020_2545_MOESM7_ESM.docx]

| **Cut off values in each dataset** | | |
| --- | --- | --- |
| **Dataset** | **patient (N)** | **Cut off value** |
| GSE30219 | 82 | 73.89346 |
| GSE31210 | 204 | 77.95697 |
| GSE50081 | 127 | 73.62206 |
| Combined data set | 413 | 54.94205 |
| GSE72094 | 311 | 81.10433 |
| TCGA-LUAD | 367 | 90.33667 |
