# Supplementary figures and images for: Development and validation of a robust immune-related prognostic signature in early-stage lung adenocarcinoma
Source: J Transl Med. 2020 Oct 7;18:380. doi: 10.1186/s12967-020-02545-z (PMC7542703; doi:10.1186/s12967-020-02545-z)

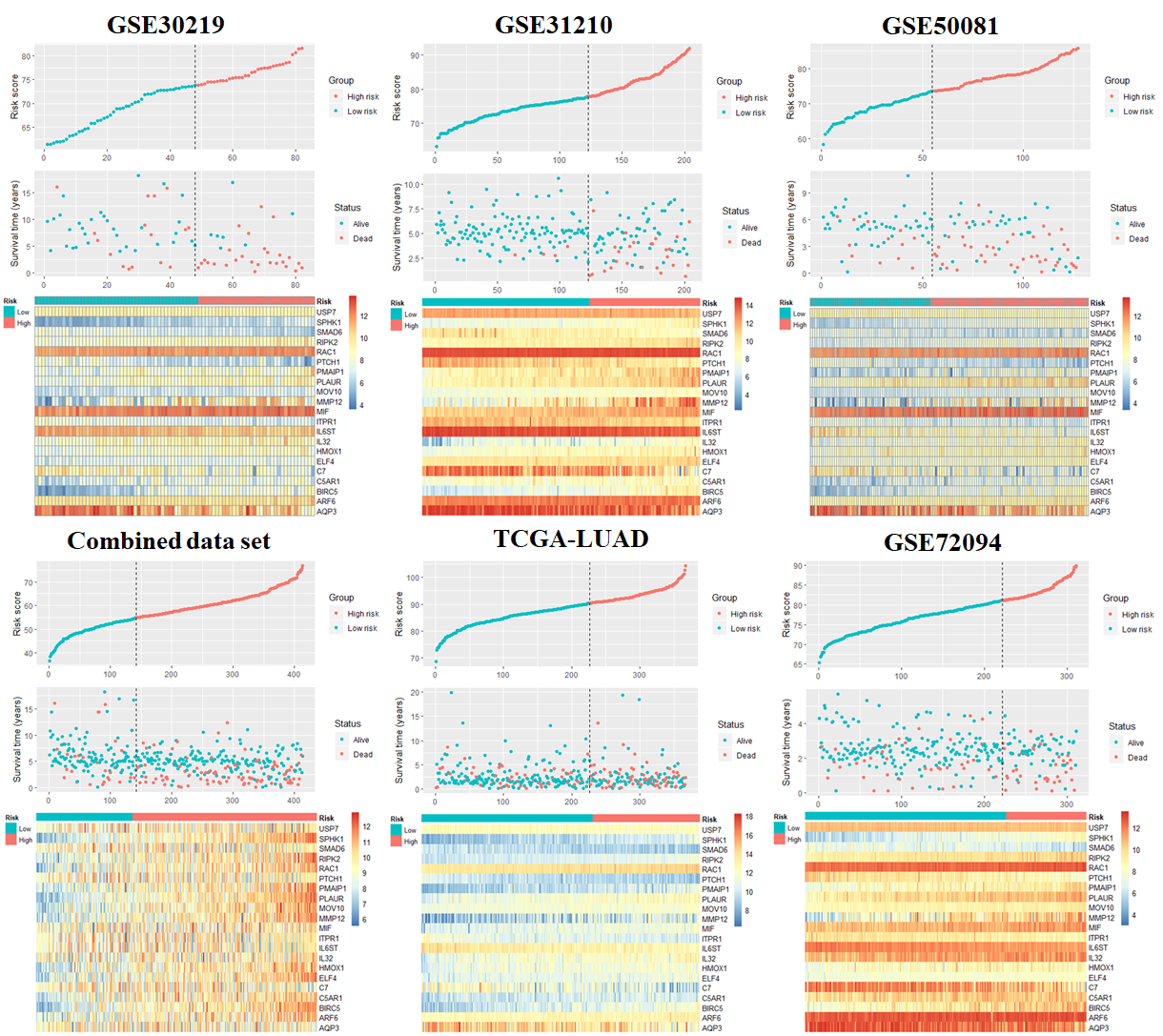

Supplement: Supplementary file 8 — Additional file 8: Figure S1. The distribution of the risk scores, survival status and gene expression levels in the enrolled data set. [file 12967_2020_2545_MOESM8_ESM.png]
